# A microfluidically controlled concave–convex membrane lens using an addressing operation system

**DOI:** 10.1038/s41378-020-0148-0

**Published:** 2020-05-18

**Authors:** Shouju Yao, Zhou Zhou, Gonghan He, Kunpeng Zhang, Xiang Huang, Bing Qiu, Daoheng Sun

**Affiliations:** 1 Fujian Micro/Nano Manufacturing Engineering Technology Research Center, Xiamen University, 361102 Xiamen, China; 2 College of Mechanical and Electrical Engineering, China Jiliang University, 310018 Hangzhou, China

**Keywords:** Applied optics, Electrical and electronic engineering

## Abstract

Electrical control toolkits for microlens arrays are available to some extent, but for applications in environments with strong electromagnetic fields, radiation, or deep water, non-electrical actuation and control strategies are more appropriate. An integrated digital microfluidic zoom actuating unit with a logic addressing unit for a built-in membrane lens array, e.g., a flexible bionic compound eye, is developed and studied in this article. A concave–convex membrane fluidic microvalve, which is the component element of the logic gate, actuator, and microlens, is proposed to replace the traditional solenoid valve. The functions of pressure regulation and decoding can be obtained by incorporating microvalves into fluidic networks according to equivalent circuit designs. The zoom actuating unit contains a pressure regulator to adjust the focal length of lenses with three levels, and the logic addressing unit contains a decoder to choose a typical lens from a hexagonal lens array. The microfluidic chip control system is connected flexibly to the actuating part, a membrane lens array. It is shown from a simulation and experimental demonstration that the designed and fabricated system, which is composed of a whole microfluidic zoom unit, addressing technology, and a microlens array, works well. Because these components are constructed in the same fabrication process and operate with the same work media and driving source, the system can be made highly compatible and lightweight for applications such as human-machine interfaces and soft robots.

## Pumping up the potential of membrane lenses

A microfluidically controlled array of membrane-based lenses could provide an effective artificial compound eye for machines operating in challenging environments. Membrane lenses can achieve adjustable focus and zoom capabilities through targeted application of contractile or compressive force. Electronic control of these lenses is not ideal for harsh conditions such as underwater or under exposure to strong radiation or magnetism, and researchers led by Zhou Zhou of China Jiliang University and Daoheng Sun of Xiamen University have devised a promising alternative. They employed a digital microfluidic system that can apply variable pneumatic pressure to individual microlenses within a compound eye structure, achieving multiple levels of zoom for each lens in a controlled and addressable fashion. These devices are straightforward to manufacture in a reproducible fashion, and could prove valuable for soft material-based robots and other applications.

## Graphical abstract

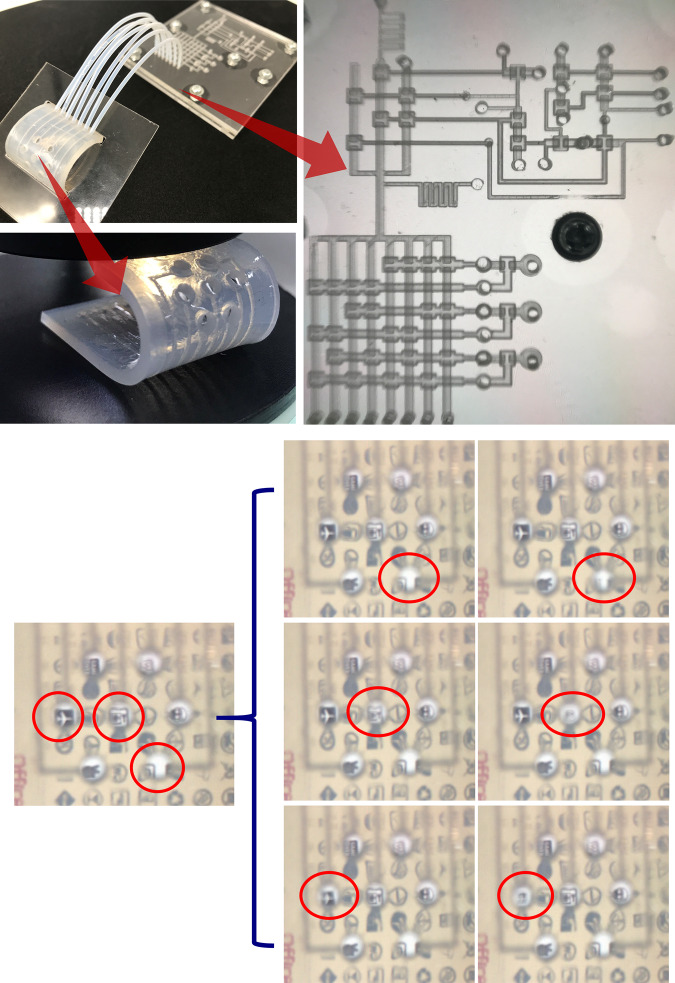

## Introduction

The optical lens is currently an important optical component in three-dimensional (3D) imaging^1^, optocouplers^2^, and miniature cell phones^3^. To meet the demand for the miniaturization and magnification of optical components, many researchers have investigated liquid lenses^4^ and membrane lenses^5^. The function of lenses is realized by the changeable surface morphology of droplets caused by electroosmosis, the deformation of a membrane directly caused by electrostatic forces, or membrane deformation caused by the uniform load of a fluid or gas medium^6^. At present, membrane lens arrays driven by electrostatic force are the most widely investigated. However, the logic control method is based mainly on signal control or solenoid valve control using the electronic circuit system. These circuit-based control methods are less responsive in water or in environments with strong electric or magnetic fields or radiation.

A typical membrane lens consists of a cavity and a sealing membrane at both ends. The zoom capability relies mainly on the deformation of a membrane under an external load. For instance, a variable-focus liquid lens was formed by applying pressure to a membrane through a piezoelectric actuator. The focal length could be adjusted from 30 mm to 500 mm under voltages between −9 and 44 V^7^. A Polydimethylsiloxane (PDMS) membrane acting as an adjustable liquid lens could also be actuated with uniform pressure applied by an electromagnetic brake. When the current was 30 mA, the maximum displacement of the membrane was 51.4 µm^8^. In addition, an integrated tuneable microlens was actuated by thermopneumatics without external pressure controllers. Its focal length ranged from 3 to 15 mm^9^. Compared to a fluidic lens, a membrane lens controlled by a pneumatic channel does not allow droplet collapse and droplet flow. This approach is ideal in 3D lens arrays to remain stable under vibration and shock.

A bionic compound eye is a typical lens array structure consisting of a collection of ommatidia that are individually controlled^10^. The control system requires good environmental adaptability and structural shape plasticity. Some studies have been conducted as follows. Zhang et al. connected multiple lenses in series to achieve simultaneous control^11^. Le et al. built a lens array on the surface of an elastic spherical shell. Simulations illustrated that the focal length of multiple lenses could be changed from 1.05 to 1.33 mm^12^. Although many studies have obtained the lens curve of artificial compound eyes^13–16^, it is still difficult to control 3D lens arrays^17^. A liquid lens array based on droplet refraction is subject to gravitational forces on the 3D curved surface, which affects the image quality. The thermal expansion force interferes with the physical fields controlled by the dielectric power. An easier method is to connect each lens to the corresponding solenoid valve and adjust the focal length through an electronic system. However, this approach requires an additional solenoid valve system, which is not conducive to the integration of future bionic systems.

Digital microfluidics is a new fluidic control technology that has emerged in recent years;^18^ similar to electronic circuits, digital microfluidics can achieve signal conditioning functions. “The first large-scale microfluidic logic network was presented by Quake in 2002^19^”. A 2n decoder circuit array based on fluidic devices was developed to construct a microfluidic analog of a comparator array and a microfluidic memory storage device. Some basic logic gates, oscillators, latches and flip-flops have been recently reported^20–24^. Based on the design of fluidic equivalent circuits, the actuating and controlling strategies of optical lenses should be more responsive. Shi et al. investigated the sorting of submicron particles using an optofluidic nanophotonic sawtooth array^25^, which illustrates that optofluidic lenses have potential applications in particle manipulation.

In this work, to meet the demand for optical zoom and addressing operations for a membrane lens array, especially for artificial compound eyes, we propose a digital microfluidic chip built in a membrane lens array. We first designed the structure of microfluidic valves and membrane lenses based on a PDMS membrane and a polymethyl methacrylate (PMMA) cavity. Then, zoom actuating units and logic addressing units based on microvalves were designed and fabricated in a microfluidic chip. Finally, the microfluidic chip control system was coupled with a membrane lens array. Experiments demonstrated the feasibility of the control method for a membrane lens array system. The digital microfluidic chips and membrane lens arrays share the same fabrication process, same work media and same driving source. The system has the advantages of compatibility and light weight, which has great potential for applications in human-machine interfaces and pneumatic soft robots.

## Results and discussion

### Microfluidic valves and logic gate

**Fig. 1:**
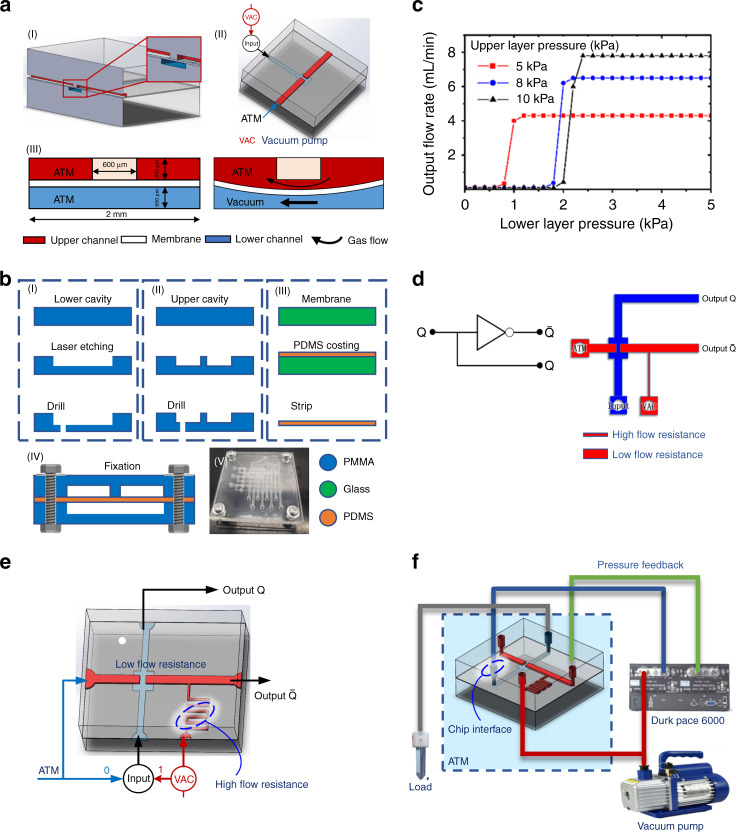
Working principle and manufacturing process of the microfluidic valve and the logic gate. **a** Working principle of a normally closed membrane microvalve. I: Three-dimensional structure of the PDMS microvalve. II: Signal input of an opened microvalve. III: Two states of a microvalve. **b** Manufacturing process of a fluidic microvalve based on laser etching. I: Manufacture of the lower flow channels and cavity. II: Manufacture of the upper flow channels and cavity. III: Manufacture of the PDMS membrane. IV: Bonding between the three layers. V: Image of the chip. **c** Switching characteristics of the microvalve. **d** Electronic logic schematic and fluidic networks of a NOT gate. **e** Signal inputs of a NOT gate. **f** External connection diagram of a NOT gate

A concave–convex membrane fluidic microvalve is the basic component of the logic gate, actuator, and microlens. Figure 1a illustrates the structure and dimensions of the PDMS (Sylgard 184, Dow Corning, USA) microvalve. It consists of an upper cavity, an intermediate PDMS membrane and a lower cavity. When the lower layer is under vacuum pressure (VAC) and the upper layer is under atmosphere pressure (ATM), the membrane moves downward. Then, the upper cavity channel is connected. At this time, the microvalve is switched from the closed state to the open state.

Due to the optical performance requirements of the system, we chose PMMA as the substrate material of the upper and lower cavities. Laser etching was used to fabricate the upper and lower structures of the microvalve. The etching process was completed by a JACV-30 engraving machine laser (Jinhuo Laser Inc, China). The laser power was 1 W, the frequency was 20 kHz, the scan interval was 20 µm and the scanning speed was 300 mms^−1^. Then, we obtained a PMMA surface etch structure with a depth of approximately 300 µm. The PDMS membrane was prepared by spin coating at 500 r min^−1^ for 1 min. After peeling from the glass substrate, the membrane had a thickness of approximately 100 μm. The three layers were bonded via thermocompression. Since the microvalve was normally closed, there was no significant pressure between the layers under negative pressure, and no additional adhesive or surface preparation was required before bonding. However, we still used screws to reinforce the three-layer structure to ensure the reliability and airtightness of the structure. The fabrication process is shown in Fig. 1b.

We investigated the switching pressure characteristics of a microvalve by connecting its outlet to the lens. The working medium in the lower layer was air, and it was water or air in the upper layer. The input pressure was sampled by a Druck Pace 6000 pressure tester (GE, USA). To ensure the processing of the flow channel structure, we designed a flow path width of 1 mm and a depth of 300 µm. Figure 1c shows the flow characteristics of a microvalve driven by different pressures. When the lower layer load pressure is greater than 1/5 of the upper layer pressure, the microvalve switches to open. Therefore, the membrane lens is subjected to a driving pressure up to five times the input pressure.

The NOT gate inverter is a logical connection method of a microvalve. As shown in Fig. 1d, a constant vacuum gas source is introduced on the side of the output, and the other side is connected to the atmosphere. In terms of bits, we define ATM as 0 and VAC as 1. When the lower layer receives an input of 0, the microvalve remains closed. Then, the constant vacuum gas flows out through the output port $\overline Q$. When the lower layer receives an input of 1, the microvalve opens. Gas at atmospheric pressure leaks through the output port *Q*, and the output $\overline Q$ is 0. At this time, a parallel flow path is produced in the lower layer, and two opposite branches are obtained.

### PDMS membrane lens

**Fig. 2:**
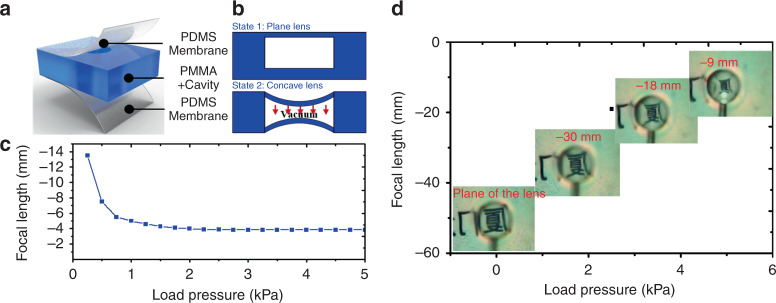
Design and optical experiment of a single PDMS membrane lens. **a** Three-layer bonded lens structure. **b** Working states of the lens. **c** Simulation curve of the focal length variation with the load pressure. **d** Deformation and imaging change in a single lens driven by pressures from −5 to 0 kPa

The lens consists of upper and lower PDMS membrane layers and a PMMA plate with a cavity in the middle (Fig. 2a). A PMMA plate with a cavity is fabricated by laser etching. When the closed cavity is subjected to vacuum pressure, the membrane is deformed inward to form a concave lens (Fig. 2b). The diameter *d* of the circular lens is 3 mm. The thickness of the PMMA plate is 3 mm, and the thickness of the PDMS membrane is 100 μm. The deformation equation of the circular membrane is^26^

$$\frac{{Pd^4}}{{16Et^4}} = \frac{{5.33w_0}}{{(1 - v^2)t}} + \frac{{2.6w_0}}{{(1 - v^2)t^3}}$$

Under the action of the load pressure *P*, the maximum deflection *w*_*0*_ at the center of a circular membrane can be obtained for a given Young’s modulus *E*, Poisson’s ratio *ν*, thickness *t* of the PDMS membrane and diameter *d*.

The curvature radius *r* of the concave membrane under vacuum pressure can be approximately modeled as a spherical shell surface. According to the maximum deflection *w*_*0*_ and the diameter *d*, we can obtain *r*. Then, in conjunction with the image focal length calculation formula for a concave lens *f* = −*r/(n*−1)^27^, as shown in Fig. 2c, we can obtain a curve of the focal length under the load pressure.

To detect the actual effect of the lens, we measured the focal length by a lens imaging experiment. We used the image of an iPad screen as a light source and observed the image in a lens under different pressures by an industrial camera (TP510, Optec Inc., USA). Figure 2d shows the deformation and imaging change in a lens driven by pressures from −5 to 0 kPa. A schematic diagram of the measurement is shown in Fig. s1.

### Microfluidic adder zoom system

**Fig. 3:**
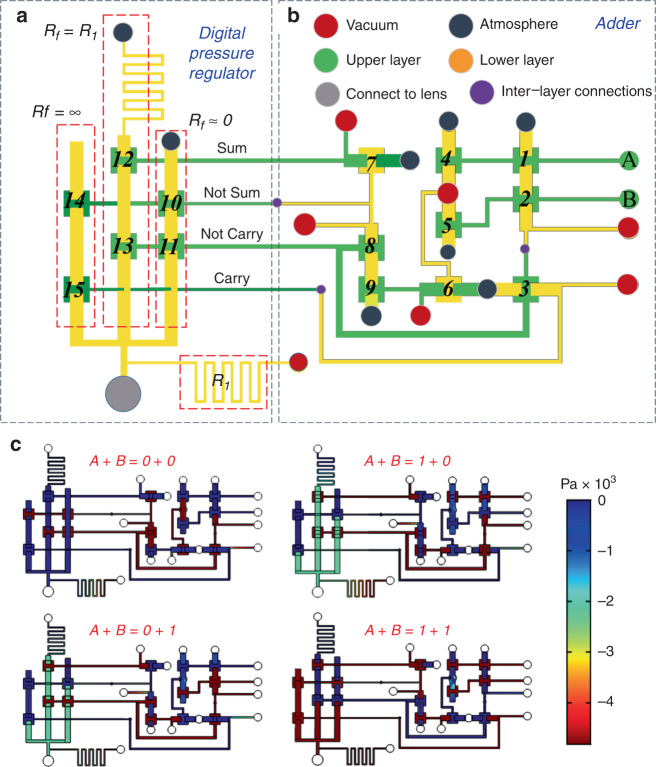
Schematic diagram of the microfluidic digital pressure regulator. **a** Digital pressure regulator. **b** Microfluidic adder logic device; A and B are input signals. **c** Simulation results of the adder logic pressure regulator

To realize the optical zoom of a membrane lens, we designed a digital pressure regulator consisting of a microfluidic adder and multichannel flow resistances, as shown in Fig. 3a. In conventional fluidic transmission systems, a pressure-reducing valve is an important device among the fluidic pressure regulating components. Here, we regard the flow resistance as the pressure relief in the pressure-reducing valve and place it between the inlet and outlet to obtain a microfluidic pressure-reducing flow path. The output pressure can be changed by altering the structure of the flow path. We distribute the channels with different flow resistance side by side, which are selected by microvalves. A digital adder is a basic arithmetic device in an electronic circuit and can achieve a desired output signal via digital accumulation. In this paper, a microfluidic adder is connected to multichannel flow resistance by microvalves to form a digital pressure regulator, which can realize the partial pressure regulating function.

An n-bit binary input can control up to 2n exit channels and can be simply understood as a microfluidic decoder. It can be controlled by an external solenoid valve system. However, solenoid valves greatly increase the size of the system when the number of input terminals is large. If 0 represents ATM and 1 represents a vacuum pressure p, we establish the following binary addition logic: 00 + 00 = 0 Pa, 00 + 01 = 01 = Δp Pa, 01 + 00 = 01 = Δp Pa, 01 + 01 = 10 = 2Δp Pa. For a 2-bit entry, when one input is a constant value and the other input is 1, the output pressure increases by Δp. If the other input is 0, the output signal is unchanged. When the original pressure of the output port is p, if it needs to be p + Δp, we input 1 to the port of the binary adder.

Microvalves 1, 2, and 3 constitute the AND gate, and the output is taken as the Carry signal of the adder. Microvalves 1, 2, 4, 5, 6, and 7 constitute the XOR gate, and microvalves 4, 5, and 6 constitute the OR gate. In addition, the output signals are logically related to the NAND gate signal output by microvalves 1 and 2. With the AND operation of valves 7, 8, and 9, the final signal is output through microvalve 7, which is similar to the XOR gate logic in the circuit. At this time, the output signal is taken as the Sum signal of the adder. Finally, the four signals Sum, Not Sum, Carry, and Not Carry are removed and connected to the pressure regulation system (Fig. 3b). To verify the accuracy of the system logic, we analyzed the pressure distribution of the regulator, which is easily obtained through the two-dimensional laminar flow simulation module of COMSOL software (COMSOL Inc., Sweden). The boundary conditions were −5 kPa at the pressure inlet and open boundary at the atmospheric port. Triangular meshes were used in the grid, and the maximum mesh size was 50 μm. The simulation results are shown in Fig. 3c. Table 1 is the input and output truth table of the simulation results.

**Table 1:** Truth table of a pressure regulator

A	*B*	Sum	Carry	Output (kPa)
0	0	0	0	0.2
1	0	1	0	2.4
0	1	1	0	2.4
1	1	0	1	5

**Fig. 4:**
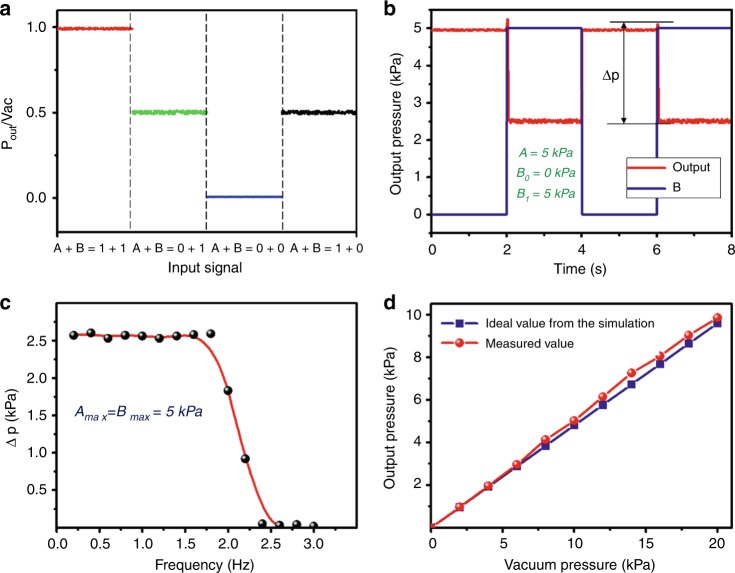
Signal characteristic curve of the pressure regulating system of a membrane lens. **a** Steady-state characteristics of the output with different input signals. **b** Output dynamic curve with a change in B. **c** System frequency response characteristics. **d** Actual and theoretical deviations of the output value

As shown in Fig. 4a, when the signals of A and B are different digital signals, the maximum of the output pressure is slightly lower than the input pressure value due to the gas leakage of the chip on the multilayer bonding surface and the interface. When A + B = 0 + 0, the output pressure is slightly larger than 0 kPa due to the slight flow resistance in the exit flow channel. The pressure of the input is measured in the steady state without considering its switching state. As shown in Fig. 4b, we fix signal A to 1, and the other input port B is switched between 0 and 1 by a solenoid valve. Then, we obtain a dynamic curve of the output pressure. During the switching process, the transient pressure increases and then declines to equilibrium because of the microvalve membrane movement caused by the gas disturbance. We regard the maximum and minimum of the output Δp as measurements to investigate the frequency characteristics of the pressure regulating system. As shown in Fig. 4c, input A is 0, and B is switched between 0 and 1. When the switching frequency of input B is lower than 2 Hz, Δp in the output remains stable. However, Δp gradually decreases when it is between 2 and 2.5 Hz because the microvalve cannot be completely closed or opened. When the switching frequency is higher than 2.5 Hz, the microvalve always maintains the initial closed state, and the output is a constant vacuum value. As shown in Fig. 4d, we measure the output pressure of the adder when A + B = 0 + 1. In the simulation results, the output pressure is half of the vacuum pressure. However, the output pressure is slightly larger in the actual measurement, and as the input pressure increases, the difference is magnified. Since protrusions in the microvalve generate a vortex under high pressure, this configuration improves the flow resistance at the outlet and affects the actual output pressure.

**Fig. 5:**
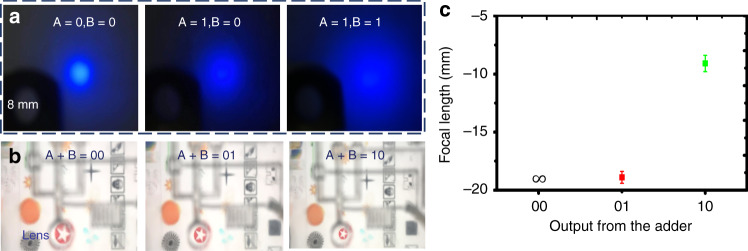
Optical performance of a membrane lens driven by a logic pressure regulator. **a** Scattering projection of the lens for different inputs. **b** Imaging of the lens for different inputs. **c** The image focal length curve for different input digital signals

In the light scattering experiment, we placed the lens between a light source and a curtain at the back of the lens at 30 mm. We selected three points with the same brightness to evaluate the change in the spot and observed it by an industrial camera (Fig. 5a). The points A + B = 0 + 0, A + B = 0 + 1, and A + B = 1 + 1 were tested, and the changes in the imaging range were analyzed under the same conditions. The corresponding output digital signals from the adder were 00, 01, and 10, and the output pressure values were 0, −2.4, and −5 kPa. As shown in Fig. 5b, the lens significantly reduced the image, but local distortion occurred. Experiments have shown that when the membrane load pressure is −5 kPa, the spot radius increases by 6 mm. Accordingly, the focal length in this case is approximately 10 mm (Fig. 5c), which is greater than the calculation result. Table s1 shows microscope readings when measuring focal length at points A + B = 01 and 10. There is a large spherical aberration in a lens because the membrane is not deformed to form an ideal spherical surface.

### Address logic for a lens array

**Fig. 6:**
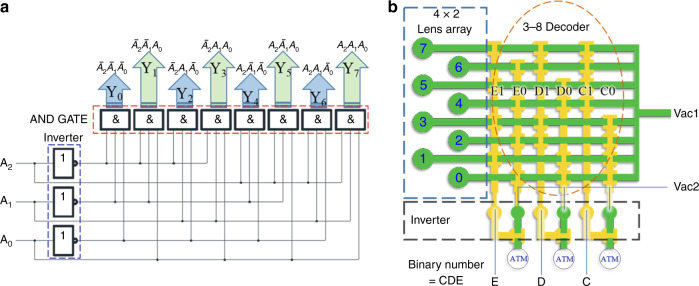
Schematic diagram of the address logic for a lens array. **a** Circuit logic structure of the decoder. **b** Schematic diagram of the integration of a lens array and a digital microfluidic network

To realize the addressing operation for a lens array, we proposed a microfluidic decoder based on the scheme of binary to decimal fluidic equivalent circuits. One of the advantages of this approach is that it simplifies the control system of a lens array. The logic schematic and microfluidic network of the decoder is shown in Fig. 6. The system consists of three parts: a microfluidic decoder, a fluidic NOT gate inverter and a lens array. The 3–8 decoding structure can control 8 output ports through 3 input ports. The same longitudinal microvalves are controlled by the same inverter output signal. The microvalve of the same lateral direction constitutes a three-position AND gate. When all the microvalves are opened, the lenses of the row are deformed by the vacuum pressure load, while the lenses of the other rows are kept in a planar state. C, D, and E are the signal input ends of the microfluidic chip, representing three binary digits. When the input signal *Q* passes through the NOT gate inverter, the signal is decomposed into two branches: *Q* and $\overline Q$. The microfluidic decoder uses the output of a three-pair NOT gate inverter as an input. The input 3-digit binary number is converted to a decimal output by the microvalve array. For example, when CDE is 011, the output of the inverters is as follows: C0 = 1, C1 = 0, D0 = 0, D1 = 1, E0 = 0, and E1 = 1. At this time, the microvalves of the channel for the lens No. 3 are all opened. The membrane under vacuum pressure is recessed downward to form a planoconcave lens.

**Fig. 7:**
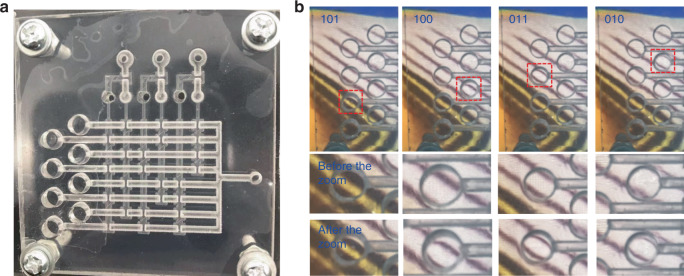
The lens array driven by a digital microfluidic chip. **a** Image of the integrated chip. **b** Image of the lenses driven by a series of digital pressure inputs

The change in lens imaging can determine whether the membrane is loaded. We connected the pipeline of the decoder to a vacuum pump, and the vacuum pressure was −5 kPa. The Vac1 terminal of the chip was connected to a constant-pressure vacuum pump. In the binary Carry order, 000–111 were input to ports C, D, and E. Finally, the working states of the lens array under different inputs were obtained by a camera. The result is shown in Fig. 7, revealing that the lenses in a lens array were operated in sequence, which verifies the feasibility of the addressing operation of a microfluidic decoder.

### Integrated control system for a flexible lens array

**Fig. 8:**
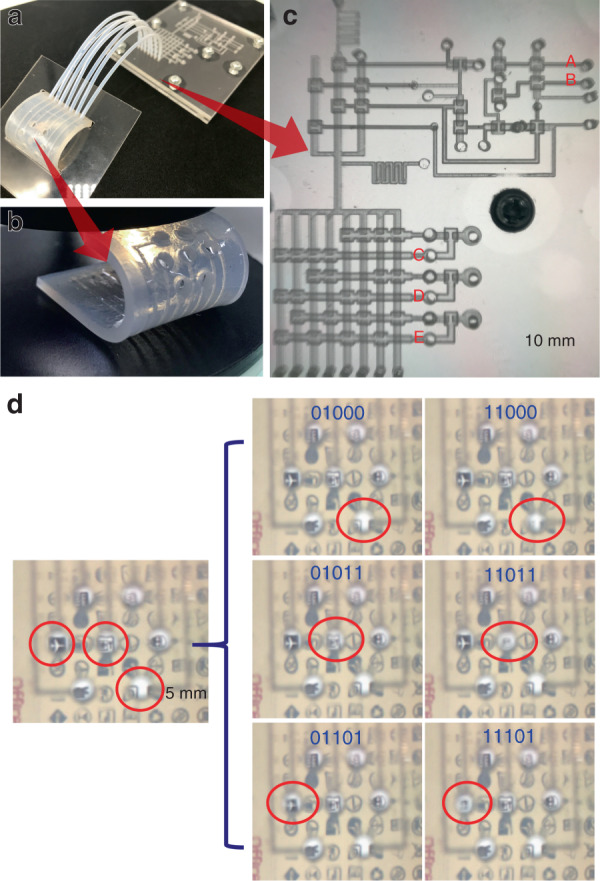
Integrated chip for the control of a compound eye, similar to a flexible lens array. **a** Image of the integrated control system. **b** Image of the flexible lens array. **c** The microfluidic structure and its pipe connection. **d** Zoom and addressing experiment with different input signals

A simple control method of lenses in a lens array is the basic requirement for a synthetic compound eye^28^. A pressure regulator and a decoder are integrated into a microfluidic chip to realize the zoom capability and addressing of a flexible lens array. Figure 8a is a diagram showing the connection of a microfluidic chip and a flexible lens array by tubing. The outlet of a pressure regulator is connected to the Vac1 port of a microfluidic decoder. As shown in Fig. 8b, the flexible lens array uses silica gel as the substrate. The fabrication of the cavity and channel structures is performed by laser etching. The cavity and the open surface of the flow channel are covered with a 100-μm PDMS membrane. According to the actual distribution law of a biological compound eye^29^, the seven lenses are arranged in a hexagonal shape with an adjacent lens distance of 5 mm. The input signal of the whole system is a 5-bit binary number, where the first two digits are the zoom system signal and the last three digits are the addressing signal. As shown in Fig. 8d, taking ABCDE = 01000 as an example, when the signals AB are changed to 11, the imaging of lens No. 0 becomes fuzzy. When the addressing signals CDE are changed to 010, the imaging of lens No. 2 becomes blurred, and lens No. 0 returns to the initial state. This experimental result is shown in movie 1.

## Conclusions

In summary, a digital microfluidic method is proposed to effectively simplify the control system of a pneumatic membrane microlens array. In this article, a digital microfluidic chip built in a membrane lens array is designed, fabricated, and characterized. Concave–convex membrane microvalves and lenses, the basic components, are proposed and analyzed. Pressure regulators and decoders, which are established by microvalves, are integrated into a microfluidic chip using a 5-bit binary number as an input to realize multilevel zoom and addressing operations. Experiments illustrate that a pressure regulator can output three levels of pressure to adjust the focal length, and a fluidic decoder can choose an operating lens from a hexagonal lens array. The switching frequency of the microfluidic system based on microvalves can reach up to 2.5 Hz under a pressure of −5 kPa. Experiments verify the feasibility of the zoom and addressing operation by connecting the microfluidic chip with a flexible lens array. A microfluidic chip can be made from various materials, such as PDMS, with high flexibility, transmittance and biocompatibility. The fluidic lens array can be coupled with the compound eye system of pneumatic soft robots.

## Materials and methods

### Materials

PDMS Sylgard 184 was obtained from the Dow Chemical Company in China. The PMMA board was obtained from Suqian Unite Acrylic Co. Ltd. and was 10 mm × 10 mm × 3 mm in size. The connection tubing was obtained from Ruixiang Silica Co. Ltd., and had an outer diameter of 1.47 mm.

### Preparation of the PDMS membrane

The PDMS membrane was fabricated according to the common coating process. The ratio of prepolymer to curing agent was 10:1. The mixture was thoroughly stirred and then degassed for 10 min in a vacuum box at −100 kPa. The coating parameters were 500 *r* min^−1^ for 60 s using a KW-4A spin coater (Chinese Academy of Sciences). To ensure the elasticity of the membrane, curing parameters of 2 h and 60 °C were used.

## Supplementary information

Supplementary information

Zoom and addressing operation
