# Supplementary material for: A microfluidically controlled concave–convex membrane lens using an addressing operation system
Source: Microsyst Nanoeng. 2020 May 18;6:34. doi: 10.1038/s41378-020-0148-0 (PMC8433168; doi:10.1038/s41378-020-0148-0)
Supplement: Supplementary file 1 — Supplementary information [file 41378_2020_148_MOESM1_ESM.docx]

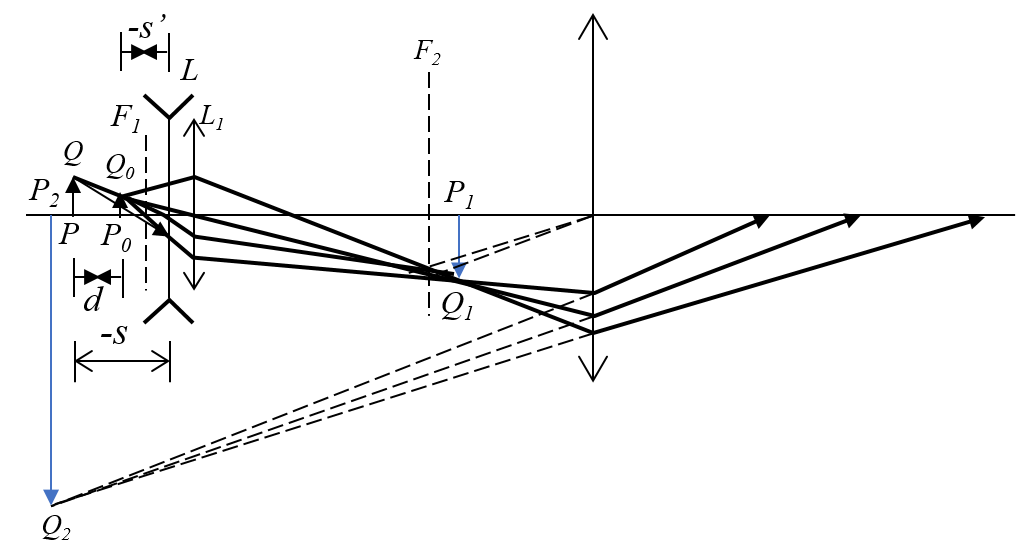


**Figure s1.** Measuring schematic diagram of the focal length of concave lens by the reading microscope. *F_1_* is the focal plane of the objective lens. *F_2_* is the focal plane of the eye lens. First, we adjust the reading microscope to obtain clear image, and record the reading. Second, we put the single lens *L* between the microscope and the screen. We record the reading by adjusting the reading microscope to obtain clear image again. According to the schematic diagram, we can calculate the focal length of the lens by the relation between object distance and image distance. According to this schematic, we can measure the object distance *s* and the movement distance of the microscope *d*, then we can calculate the image distance *s’*, which is *s-d*. Finally, we obtain the focal length by the formula *1/s’-1/s=1/f*.

**Table s1.** The readings of the microscope.

| A+B | Screen position (mm) | Lens position (mm) | *d_1_*(mm) | *d_2_* (mm) | *d*(mm) | *s* (mm) | *f* (mm) |
| --- | --- | --- | --- | --- | --- | --- | --- |
| 01 | 8 | 22.5 | 75 | 81.3 | 6.3 | 14.5 | 18.9 |
| 10 | 8 | 22.5 | 75 | 83.9 | 8.9 | 14.5 | 9.12 |
